# Neurovascular coupling methods in healthy individuals using transcranial doppler ultrasonography: A systematic review and consensus agreement

**DOI:** 10.1177/0271678X241270452

**Published:** 2024-08-07

**Authors:** James D Ball, Eleanor Hills, Afzaa Altaf, Pranav Ramesh, Matthew Green, Farhaana BS Surti, Jatinder S Minhas, Thompson G Robinson, Bert Bond, Alice Lester, Ryan Hoiland, Timo Klein, Jia Liu, Nathalie Nasr, Rehan T Junejo, Martin Müller, Andrea Lecchini-Visintini, Georgios Mitsis, Joel S Burma, Jonathan D Smirl, Michael A Pizzi, Elsa Manquat, Samuel JE Lucas, Karen J Mullinger, Steve Mayhew, Damian M Bailey, Gabriel Rodrigues, Pedro Paulo Soares, Aaron A Phillips, Prokopis C Prokopiou, Lucy C Beishon

**Affiliations:** 1 Department of Cardiovascular Sciences, 4488 University of Leicester, Leicester, UK; 2 NIHR Leicester Biomedical Research Centre, British Heart Foundation Cardiovascular Research Centre, Glenfield Hospital, Leicester, UK; 3 Public Health and Sports Sciences, University of Exeter Medical School, University of Exeter, Exeter, UK; 4 Division of Critical Care Medicine, Department of Medicine, Faculty of Medicine, Vancouver General Hospital, University of British Columbia, Vancouver, BC, Canada; 5 Division of Neurosurgery, Department of Surgery, Faculty of Medicine, University of British Columbia, Vancouver, BC, Canada; 6 Institute of Sports Science, University of Rostock, Rostock, Germany; 7 Shenzhen Institutes of Advanced Technology, Chinese Academy of Sciences, Shenzhen University Town, Shenzhen, China; 8 Department of Neurology, Poitiers University Hospital, Poitiers, France; 9 Department of Life Sciences, Manchester Metropolitan University, Manchester, UK; 10 Department of Neurology and Neurorehabilitation, Lucerne Kantonsspital, Spitalstrasse, Lucerne, Switzerland; 11 School of Electronics and Computer Science, University of Southampton, Southampton, UK; 12 School of Bioengineering, McGill University, Montreal, Quebec, Canada; 13 Sport Injury Research Prevention Center, University of Calgary, Calgary, Alberta, Canada; 14 Department of Neurology, University of Florida, Florida, USA; 15 Department of Anaesthesia and Critical Care, Hospital Lariboisière, Assistance Publique des Hôpitaux de Paris, Paris, France; 16 School of Sport, Exercise and Rehabilitation Sciences & Centre for Human Brain Health, University of Birmingham, Birmingham, UK; 17 Sir Peter Mansfield Imaging Centre, School of Physics and Astronomy, 6123 University of Nottingham, Nottingham, UK; 18 School of Psychology, Aston University, Aston, UK; 19 Faculty of Life Sciences and Education, University of South Wales, Pontypridd, UK; 20 Department of Clinical Sciences and Community Health, 9304 University of Milan, Milan, Italy; 21 Department of Physiology and Pharmacology, Fluminense Federal University, Niterói, Brazil; 22 Department of Physiology and Pharmacology, University of Calgary, Alberta, Canada; 23 Athinoula A. Martinos Center for Biomedical Imaging, Massachusetts General Hospital, Charlestown, MA, USA; 24 Department of Radiology, Harvard Medical School, Boston, MA, USA

**Keywords:** Healthy, narrative summary, neurovascular coupling, systematic review, transcranial Doppler ultrasonography

## Abstract

Neurovascular coupling (NVC) is the perturbation of cerebral blood flow (CBF) to meet varying metabolic demands induced by various levels of neural activity. NVC may be assessed by Transcranial Doppler ultrasonography (TCD), using task activation protocols, but with significant methodological heterogeneity between studies, hindering cross-study comparisons. Therefore, this review aimed to summarise and compare available methods for TCD-based healthy NVC assessments. Medline (Ovid), Scopus, Web of Science, EMBASE (Ovid) and CINAHL were searched using a predefined search strategy (PROSPERO: CRD42019153228), generating 6006 articles. Included studies contained TCD-based assessments of NVC in healthy adults. Study quality was assessed using a checklist, and findings were synthesised narratively. 76 studies (2697 participants) met the review criteria. There was significant heterogeneity in the participant position used (e.g., seated vs supine), in TCD equipment, and vessel insonated (e.g. middle, posterior, and anterior cerebral arteries). Larger, more significant, TCD-based NVC responses typically included a seated position, baseline durations >one-minute, extraneous light control, and implementation of previously validated protocols. In addition, complementary, combined position, vessel insonated and stimulation type protocols were associated with more significant NVC results. Recommendations are detailed here, but further investigation is required in patient populations, for further optimisation of TCD-based NVC assessments.

## Introduction

Neurovascular coupling (NVC) is the process ensuring metabolic demands induced by neuronal activity are met via rises in cerebral blood flow (CBF). This is coordinated by the neurovascular unit (NVU),^1^ consisting of vascular smooth muscle, neurons, pericytes and astrocytes mediating NVC responses proportionally to cognitive demand.^2^ This coordinated activity, induced by various processes including those that are endothelium-specific processes, dilates arteries and arterioles,^3^ primarily through three mechanisms: metabolic, sheer wall stress and autonomic.^4–6^

Metabolic CBF regulation is mediated by “feedback” and “feedforward” mechanisms.^1^ “Feedback” is triggered by metabolic activity inducing vasoactive metabolite (e.g., lactate and nitric oxide) release, causing vasodilation and CBF increases. The glutamate-mediated “feedforward” mechanisms include neuronal synaptic activity causing prostanoid and mediator (e.g., K^+^) release.^4^ These vasoactive biproducts regulate CBF, differing from “feedback” which is driven by synaptic activity and is not dependent on tissue metabolic state.^4^

The shear wall stress mechanism prevents vascular damage due to increased CBF.^4,5^ Raised CBF increases pressure on vessel walls causing shear wall stress. This releases vasoactive mediators (K^+^), inducing vasodilation. Autonomic mechanisms regulate NVC through adjusting sympathetic/parasympathetic tone to modulate vascular smooth muscle contraction.^4,5^ Parasympathetic nerve fibres innervate cerebral blood vessels, which induce vasodilation when activated.^6^

**Figure 1.**
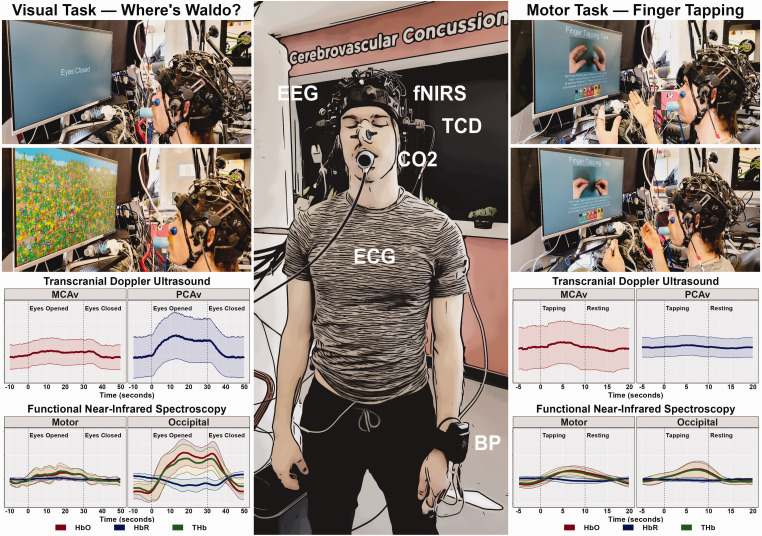
Representative TCD-based NVC assessment set-up and results. The centre panel display a representative photo of an individual wearing a multimodal headcap consisting of bilateral transcranial Doppler ultrasound (TCD), functional near-infrared spectroscopy (fNIRS) (an optional multimodal addition to TCD), and electroencephalography (EEG) with peripheral monitoring for systemic influences of carbon dioxide (CO_2_) via capnography, heart rate (HR) via electrocardiography (ECG), and blood pressure (BP) via finger photoplethysmography corrected for heart level. The top left panels demonstrate an individual completing a visual “Where’s Waldo?” task with both eyes closed and eyes opened instructions. The bottom left panels display the representative middle cerebral artery velocity (MCAv) and posterior cerebral artery velocity (PCAv) during the visual task via TCD and the oxygenated haemoglobin (HbO), deoxygenated haemoglobin (HbR), and total haemoglobin (THb) responses via fNIRS in the motor and occipital cortices. The top right panels demonstrate an individual completing a visually-cued motor finger tapping task during period of rest and active motor engagement. The bottom right panels display the representative MCAv and PCAv responses during the motor task via TCD and the HbO, HbR, and THb responses via fNIRS in the motor and occipital cortices.

Several neuroimaging techniques indirectly measure NVC including Transcranial Doppler ultrasonography (TCD), functional near infrared spectroscopy (fNIRS), functional magnetic resonance imaging (fMRI), positron emission tomography (PET), and single-photon emission computerized tomography (SPECT).^7^ TCD is a portable, non-invasive technique using ultrasound to measure cerebral blood velocity (CBv) in the anterior, middle, or posterior cerebral arteries (ACA, MCA & PCA, respectively). CBv acts as a proxy for CBF, assuming vessel diameter, blood pressure (BP) and CO_2_ remain constant.^7^ TCD is safe and typically tolerable for participants.^8^ An example set up for a TCD-based NVC assessment is presented in Figure 1. fNIRS uses infrared light (780–2500 nm) to measure changes in cerebral blood haemoglobin oxygenation.^8^ fNIRS is also portable and non-invasive but is only capable of measuring superficial changes in the cortex. TCD and fNIRS have good temporal but limited spatial resolution.^8^ fMRI uses blood oxygen level-dependent magnetic resonance imaging (BOLD-fMRI) to assess NVC, with better spatial, but poorer temporal resolution.^9^ BOLD-fMRI does not give absolute CBF measurements. Furthermore, fMRI is expensive, technically challenging, and less well tolerated, particularly in those with cognitive impairment.^10^ PET and SPECT use radiolabelled tracers to measure glucose uptake in high metabolic activity areas. However, false positives and negatives are common.^11^ PET and SPECT are also expensive options, require technical expertise, and use ionising radiation, limiting their widespread use. However, TCD has limitations such as ∼10–15% of people lacking adequate insonation windows, but this will vary dependant on populations studied and techniques used.^7^

NVC is assessed through stimulation protocols, inducing neural activity and corresponding CBF rises, including cognitive, motor, auditory and visual paradigms.^12,13^ An example NVC response to cognitive stimulus using TCD is provided in Figure 1. Due to the range of imaging techniques and stimulation protocols in NVC-based research, there is significant methodological heterogeneity to evoke NVC responses, and a lack of consensus regarding optimal NVC study methods. This has led to conflicting clinical study results. One example is age-related NVC changes, with contradictory increased, decreased, and neutral CBF/CBv age-related changes reported.^13–15^ Similarly, there is contradiction among the effects of sex on NVC responses. For example, Burma et al.,^16^ found differences in NVC responses between males and females were related to task engagement. However, Leacy et al.,^17^ showed visually evoked NVC responses are neither age nor sex dependent. It remains unclear whether discordant study findings stem from physiological, populational, methodological or analytical variation, particularly amongst studies using TCD-based stimulation protocols.

Therefore, this review sought to summarise available literature on methods used to conduct TCD-based NVC studies in healthy populations. We aimed to summarise types of stimulation, study designs, protocol, and analysis characteristics. Also, we identified areas of heterogeneity in methods and protocols, providing suggestions to improve between-centre consistency through consensus with experts conducting TCD-based NVC research internationally.

## Methods

This review is reported in line with the Preferred Reporting Items for Systematic Reviews and Meta-Analysis (PRISMA) guidance on reporting systematic reviews.

### Database searches and screening

Medline, Scopus, Web of Science, PsychINFO, EMBASE and CINAHL were searched in Ovid from inception to 14/12/2022 with strategy provided (Supplementary Appendix 1), limited to healthy, human studies published in English. The protocol for the review was pre-registered on PROSPERO (CRD42019153228). The search strategy was developed in conjunction with a librarian (Supplementary Appendix 1). Duplicates were removed and initial screening was conducted using Covidence, and subsequently in Rayyan (Qatar Computing Research Institute).^18^ Articles were screened on title/abstract independently by five reviewers (all were screened by at least one reviewer for consistency – LB). Articles were also screened at full text by three reviewers (JB, EH and AA). Disagreements at title and abstract were arbitrated by LB and JB.

### Inclusion and exclusion criteria

Articles were screened against the following inclusion criteria: adults aged ≥18 years, NVC outcome present (e.g., percentage change in CBv), healthy participants, TCD-based protocol. All study designs were considered for inclusion: randomised and non-randomised controlled trials, quasi-experimental studies, case-control studies, validation, or reproducibility studies using TCD to investigate NVC, including multi-modal studies also employing other imaging modalities (e.g., fNIRS, MRI). Exclusion criteria included persons aged <18 years, no measure of cerebral haemodynamics, patient/disease groups, no outcome measure of NVC or no use or mention of TCD within title and/or abstract/focus on other imaging modalities.

### Quality assessment

Study quality was evaluated using pre-defined criteria used previously by this group (Supplementary Appendix 2).^19^ Quality assessment was completed independently by JB and four review authors (AA, EH, MG and PR). Disagreements were resolved by discussion/arbitrated by a third review author, where necessary (LB). Overall quality scores/15 were given to each study, as summarised in Table 1.

**Table 1.** Summary of characteristics of included studies.

Author, year	Participant position	Baseline Duration	Stimulation Duration	Recovery Duration	Stimulation Type	Vessel Insonated	Quality Assessment Score	DOI
Azevedo, 2007	Seated/Standing	NR	40 s	20 s	Visual	PCA and MCA	13	doi.org/10.1007/s00415-006-0338-1
Bäcker, 1999	Seated	15 s	5 s	NR	Cortical tuning	MCA	10	doi.org/10.1097/00001756-199902050-00016
Bader, 2021	Seated	1 m	30 s	30 s	Visual	PCA	12	doi.org/10.14814/phy2.14952
Balogh, 2019	Seated	5 s	40 s	20 s	Visual	PCA	14	doi.org/10.1002/jcu.22702
Beishon, 2017	Seated	5 m	Various (ACE-III)	30 s	Attention, memory, fluency, language, visuospatial	MCA	13	doi.org/10.1016/j.jneumeth.2017.08.019
Beishon, 2018	Seated	5 m	Various (ACE-III)	30 s	Attention, memory, fluency, language, visuospatial	MCA	14	doi.org/10.1152/jn.00698.2017
Beishon, 2018	Seated	5 m	Various (ACE-III)	30 s	Attention, memory, fluency, language, visuospatial	MCA	14	doi.org/10.14814/phy2.13803
Beishon, 2018	Seated	5 m	Various (ACE-III)	30 s	Attention, memory, fluency, language, visuospatial	MCA	14	doi.org/10.2174/1874609812666190131165310
Beishon, 2020	Seated	5 m	30 s	30 s–60 s	Attention, memory, fluency, language, visuospatial	MCA	13	doi.org/10.1016/j.jneumeth.2020.108779
Beishon, 2021	Seated	5 m	2 m	1 m	Cognitive	MCA	14	doi.org/10.3233/jad-201444
Bliss, 2022	Seated	30 s	6 m/various	5 m	Motor/cognitive	MCA	14	doi.org/10.3389/fnagi.2022.892343
Boban, 2014	Seated	180 s	∼30 s	60 s/120 s	Cognitive	ACA	11	doi.org/10.1016/j.bandc.2013.10.006
Bogdanova, 2015	NR	60 s	Various	60 s	Cognitive	MCA PCA	6	doi.org/10.17759/exppsy.2015080306
Bracco, 2011	Seated	2 m	Various	2 m between different tasks	Visual cognitive tasks	MCA	11	doi.org/10.1016/j.cortex.2010.03.007
Bulla-Hellwig, 1996	NR	60 s	Various	60 s	Cognitive	MCA PCA	8	doi.org/10.1016/0028-3932(96)00021-8
Bullock, 2021	NR	15 s	500–750 ms	45 s	Visual/cognitive	MCA and PCA	12	doi.org/10.14814/phy2.15106
Burma, 2021	Seated	5 s	40 s	20 s	Visual	MCA and PCA	14	doi.org/10.1177/0271678x221084400
Burma, 2021	Seated	5 s	40 s	20 s	Visual	MCA and PCA	14	doi.org/10.14814/phy2.14695
Burma, 2022	Seated	5 s	40 s	20 s	Visual	MCA and PCA	14	doi.org/10.14814/phy2.15020
Caldwell, 2017	NR	2 m	30 s	30 s	Visual	PCA and MCA	13	doi.org/10.1016/j.physbeh.2018.02.035
Caldwell, 2018	NR	5 m	30 s	30 s	Visual	PCA and MCA	12	doi.org/10.1016/j.physbeh.2017.09.023
Caldwell, 2021	Seated	2 m	30 s	30 s	Visual	MCA and PCA	13	doi.org/10.1139/apnm-2020-0562
Castro, 2012	Seated/Standing/Head-up tilt	NR	40 s	20 s	Visual	PCA and MCA	13	doi.org/10.1016/j.permed.2012.02.052
Cilhoroz, 2022	Seated	10 s	3 m	4 m	Visual/Cognitive	MCA	14	doi.org/10.3389/fcvm.2022.914439
Csipo, 2021	Seated	100 s	250 ms	2000 ms	Visual	MCA	12	doi.org/10.1371/journal.pone.0250043
Csipo, 2021	Seated	250 ms	250 ms	2000 ms	Visual	MCA	12	doi.org/10.1038/s41598-021-00188-8
Duschek, 2008	Seated	10 s	5 s	55 s	Visual/audio	MCA	11	doi.org/10.1111/j.1469-8986.2010.01020.x
Duschek, 2008	NR	60 s	60 s	60 s	Cognitive	MCA	11	doi.org/10.1016/j.clinph.2008.01.102
Duschek, 2010	NR	3 m	5 s	55 s	Visual/audio	MCA	12	doi.org/10.1027/0269-8803.22.2.81
Evans, 2017	Seated	NR	Various	NR	Cognitive	MCA	14	doi.org/10.3390/nu9010027
Fitri, 2018	NR	60 s	Various - Stroop Test	Various	Stroop test - Victoria version	ACA	12	doi.org/10.1088/1755-1315/125/1/012203
Haase, 2005	NR	NR	15s	5 s	Verbal memory	MCA	12	doi.org/10.1016/j.pnpbp.2005.01.006
Hartje, 1994	Supine	60 s	Various	60 s	Cognitive	MCA	7	doi.org/10.1016/0028-3932(94)90116-3
Heffernan, 2015	Walking	15 m	4 m	4 m	Cognitive perturbation	MCA	14	doi.org/10.1093/ajh/hpu198.
Intharakham, 2022	Seated	30 s	5 s, 30 s and 60 s	60 s	Visual/Cognitive	MCA	12	doi.org/10.1371/journal.pone.0266048
Jor’dan, 2017	Seated/Walking	300 s	4 s	5 s	Visual	L/R MCA	13	doi.org/10.1093/gerona/glx063.
Jor'dan, 2021	Seated/Walking	250 ms	3 s	2 s	Visual/cognitive	MCA	12	doi.org/10.1093/gerona/glz006
Kelley, 1992	NR	NR	NR	NR	Visual/cognitive	ACA, MCA, PCA	10	doi.org/10.1161/01.str.23.1.9.
Klingelhöfer, 1997	Seated	30 s	30 s (50 s memory test)	30 s	Motor/spatial/audio/memorization trials/recognition	L/R MCA	10	doi.org/10.1097/00004647-199705000-000
Knecht, 1996	Seated/Supine	60 s	15 s	60 s	Visual/audio/motor	MCA	10	doi.org/10.1097/00001756-199602290-00033.
Ladthavorlaphatt, 2022	Seated	5 m	60 s/30 s/5 s	60 s	Visual/Cognitive	MCA	14	doi.org/10.1016/j.medengphy.2022.103921
Leacy, 2022	Seated	10 m	30 s	30 s	Visual	PCA	13	doi.org/10.1152/japplphysiol.00292.2021
Lefferts, 2015	Supine	10 m	4 m (Stroop color-word interference task)	NR	Visual, motor	MCA CCA		doi.org/10.1152/japplphysiol.00100.2018.
Lefferts, 2018	Supine	10 m	180 s	60 s	Visual	MCA	13	doi.org/10.1139/apnm-2015-0400
Lefferts, 2020	Seated	60 s	180 s	NR	3-minute incongruent Stroop task	MCA ACA	12	doi.org/10.1089/ham.2019.0050
Madureira, 2017	Supine	10 m	4 s	5 s	Visual	MCA	13	doi.org/10.1016/j.jstrokecerebrovasdis.2016.12.003
Matteis, 1997	Supine	2 m	30 s/120 s	120 s	Audio	MCA	10	doi.org/10.1016/s0022-510X(97)05375-6
Merchant, 2017	Supine	16 m	4 s	5 s	Visual	MCA	13	doi.org/10.1152%2fajpheart.00438.2016
Meyer, 2014	NR	5 s	25–35 s	15–25 s	Language lateralisation	MCA	12	doi.org/10.3389/fpsyg.2014.00552
Misteli, 2011	NR	NR	Various (TMT)	60s between tasks	Visual, motor	MCA	12	doi.org/10.1016/j.bandc.2011.02.009
Moody, 2005	Seated	10 m	30 s	30 s	Visual and motor	MCA	14	doi.org/10.1152/ajpregu.00837.2004
Murata, 2015	Seated/Walking	NR	120 s	120 s	Cognitive	NR	12	doi.org/10.1109/hsi.2015.7170684
Nowakowska-Kotas, 2015	NR	30 s	195 s	120 s	Audio	MCA	13	doi.org/10.4103%2f1463-1741.169709
Panerai, 2005	Seated	10 m	30 s	30 s	“Brain activation tasks” visual/motor	MCA	12	doi.org/10.1152/ajpregu.00161.2012
Panerai, 2012	Seated	Average of 10 rests	30 s	30 s	Cognitive-motor paradigms	MCA	14	doi.org/10.1152/ajpheart.00115.2005
Pearson, 2021	Supine	60 s	3 m	2 m	Visual/cognitive	MCA	14	doi.org/10.1152/ajpregu.00048.2021
Roby, 2021	Seated	2 m	40 s	20 s	Visual	PCA	13	doi.org/10.1007/s10439-020-02604-y
Roby, 2021	Seated	NR	40 s	20 s	Visual/Cognitive	PCA and MCA	14	doi.org/10.1007/s10439-021-02764-5
Rosengarten, 2001	Seated	NR	40 s	20 s	Visual	PCA (left and right)	12	doi.org/10.1016/s0301-5629(01)00355-6
Shaw, 2009	Seated	5 m	125 ms/200 ms	60 s	Visual/audio	MCA	8	doi.org/10.1016/j.neulet.2009.06.008
Shaw, 2013	Seated	60 s	4 s	NR	Audio	L/R MCA	11	doi.org/10.1080/00140139.2013.809154
Smirl, 2016	Seated	NR	40 s	20 s	Visual	PCA and MCA	13	doi.org/10.1016/j.jneumeth.2016.06.007
Sorond, 2008	Supine	30/40 s	30–40 s	30–40 s	Cognitive	PCA (ACA)	11	doi.org/10.1016/j.cortex.2006.01.003
Spence, 2021	Seated	10 m	30 s	30 s	Visual	MCA and PCA	14	doi.org/10.1016/j.physbeh.2020.113198
Spronck, 2012	Seated	5 m	60 s	60 s	Visuospatial	PCA and MCA	11	doi.org/10.1152/ajpheart.00303.2012
Stefanidis, 2019	Seated	Average of 10 rests	Reading (40 s) hypocapnia (30 s analysed from 3 min of stimulation) sit to stand (every 10 s for 5 mins)	Reading (20 s rest), 5 mins between block paradigms	Visual	Left MCA	12	doi.org/10.1371/journal.pone.0217082
Stefanidis, 2020	Seated/Sit-stand	30 s	10 s / 180 s	10 s/NR	Pressure-flow and hypocapnic	MCA	11	doi.org/10.1016/j.jocn.2020.09.034
Stroobant, 2004	NR	60 s	120 s	120 s	Visuospatial and verbal	MCA	12	doi.org/10.1111/j.1468-1331.2004.00873.x.
Toth, 2022	NR	NR	60 s/45 s	60 s	Cognitive/motor	MCA	13	doi.org/10.1007/s11357-022-00623-2
Vadikolias, 2007	Seated	5 m	60 s	60 s	Language activation	MCA	11	doi.org/10.1038/sj.jcbfm.9600484
Vingerhoets, 1999	Seated	60 s	120 s	120 s	Verbal and visuospatial	MCA	10	doi.org/10.1016/s0028-3932(01)00030-6
Vingerhoets, 2001	Seated	60 s	90 s/156 s	120 s	Audio	MCA	10	doi.org/10.1161/01.str.30.10.2152
Viski, 2016	Seated	5 s	40 s	20 s	Visual	PCA and MCA	14	doi.org/10.1016/j.jns.2016.02.050
Viski, 2016	Seated	5 s	40 s	20 s	Visual / braille reading	MCA PCA	14	doi.org/10.1016/j.jns.2016.02.050
Williams, 2017	Seated	5 m	Various (ACE-III)	30 s	Attention, memory, fluency, language, visuospatial	MCA	12	doi.org/10.1016/j.jneumeth.2017.04.013
Wong, 2016	NR	10 s	30 s	30 s	Mood	MCA and PCA	11	doi.org/10.1016/j.trci.2016.07.003

NR: not reported; MCA: middle cerebral artery; PCA: posterior cerebral artery; ACA: anterior cerebral artery; L/R: left/right; ACE-III: Addenbrooke’s Cognitive Examination III; TMT: trail making test.

### Data extraction

Data were extracted into Microsoft Excel by one review author (JB). A full list of data extraction variables is provided (Supplementary Appendix 3). But broadly, data were extracted on eight categories: study characteristics, participant characteristics, preparation, assessment characteristics, protocol characteristics, measurement characteristics, analysis characteristics and reproducibility measures. 20% of extracted data was checked independently by a second reviewer (EH), as quality control. Key study characteristics have been summarised (Table 1).

### Narrative synthesis

Due to significant heterogeneity, meta-analysis was precluded, and findings were synthesised narratively. To draw recommendations for future research, an international consensus group of 24 experts was convened through the Cerebrovascular Research Network (CARNet). Each category was reviewed by experts in TCD or NVC research before review by the wider group for ratification in the final published report. The consensus group was divided into ten sections (study/participant characteristics, participant preparation, assessment characteristics, protocol characteristics, measurement characteristics, analysis characteristics, reproducibility, clinical applications, extensions/multimodal techniques, and mechanisms/physiology), and smaller groups worked on each of these sections. Each section was reviewed at a larger meeting with the consensus group. In total, the group met four times to review and agree recommendations for each section of the review. The final recommendations were reviewed by all co-authors in the final version of the manuscript.

### Protocol modifications

The original protocol included fNIRS studies and studies with both clinical and healthy populations. This yielded a large amount of literature which was not feasible to review in detail. As such, the search was narrowed to only healthy studies using TCD. We also excluded any studies conducted in a healthy population which used environmental (e.g., altitude), or drugs/interventions to modulate NVC.

## Results

### Summary of included studies

**Figure 2.**
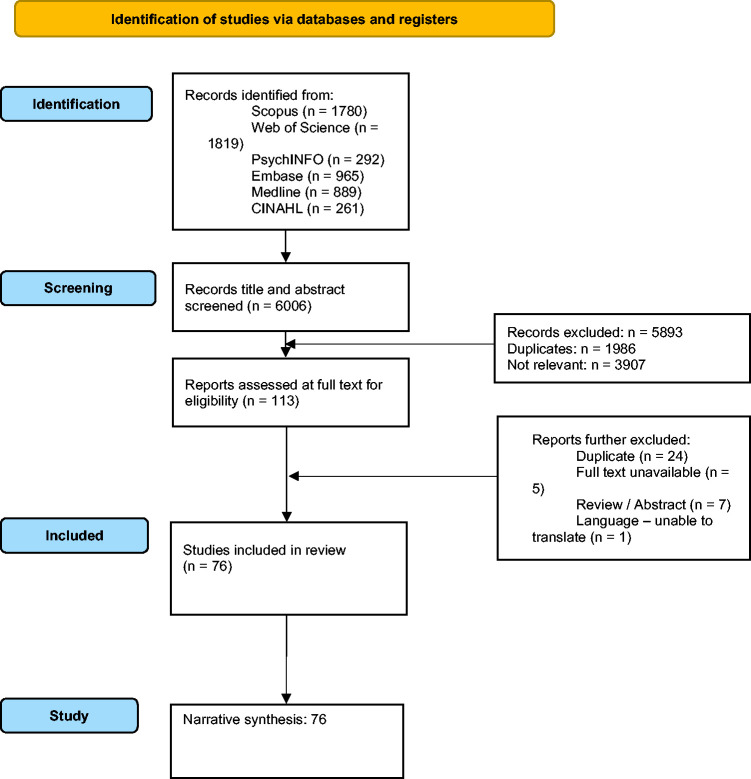
Prisma flow diagram for narrative synthesis.

6006 studies were identified from literature database searches. 1986 were duplicates, leaving 4020 articles for title/abstract screening. After title/abstract screening, 3907 were excluded, leaving 113 papers for full-text screening. Full-text screening identified a further 37 ineligible studies owing to manuscript availability or language barriers. Therefore, 76 final studies were eligible for inclusion (Table 1 and Figure 2).

### Characteristics of included studies

All 76 included studies performed TCD-based NVC assessments in 2697 healthy participants. Studies were published from 1992–2022, with 54% female participants and ages ranging 19–85 years. Eight studies also concomitantly included participants with stable and well-controlled comorbidities including hypertension and blindness. 41 studies (54%) were performed in Europe, 28 (37%) in North America, five (7%) in Australia and two (3%) in Asia. Sample sizes ranged from five to 135 participants. This review included studies which reported NVC findings in a range of formats.

### Summary of quality assessment

The median quality assessment score for all included studies was 12 with an interquartile range of 11–14 (Table 1). Predominant strengths included study publication in peer-reviewed journals and accurately described study populations (n = 76, 100%). Other strengths included clearly described study protocols and clearly presented/appropriate statistical methods (n = 75, 99%). Weaknesses included a lack of pre-determined sample size calculations (n = 10, 13%) and poorly described inclusion and exclusion criteria (n = 54, 71%).

### Preparation

Studies differed with reference to preparation of study participants and conditions. Most studies (n = 59, 78%) conducted experiments with participants in fixed positions, however, some employed multiple positions. This review identified seven postural conditions including seated (n = 51, 67%), supine (n = 12, 16%), walking (n = 4, 5%), standing (n = 3, 4%), sit-to-stand (n = 1, 1%) and 70° head-up tilt (n = 1, 1%). Seated positions were associated with larger, more significant NVC responses. One fifth of studies (n = 16, 21%) did not report participant position, hindering between-study comparisons.

Few studies reported or controlled for time of day, with only eleven studies (14%) conducting experiments at designated times. Similarly, few studies controlled for environmental light intensity (n = 19, 25%) or temperature (n = 18, 24%). However, studies that did impose study environment light control produced larger NVC responses with light-controlled showing %CBv change from 3–30%, whereas studies which did not control for environmental light ranged from non-significant change up to ∼30% increase.

More studies ensured quiet environments (n = 30, 39%) (without decibel criteria) and controlled for effects of alcohol (n = 33, 43%), but fewer (n = 25, 33%) controlled for exercise (abstention for fixed durations beforehand), or heavy meals (n = 18, 24%). However, a fasted state was not used in studies which produced the most significant NVC responses. Overall, there was significant between-study heterogeneity, even when controlling for experimental position, time of day, extraneous noise or light, and effects of alcohol, leading to variation of NVC responses induced.

### Assessment characteristics

Of the 76 included studies, most (n = 53, 70%) reported beat-to-beat BP assessments alongside CBF, but a sizeable proportion (n = 23, 30%) did not. Fewer studies assessed breath-by-breath end-tidal CO_2_ (EtCO_2_) (n = 39, 51%) and beat-to-beat heart rate (HR) (n = 51, 67%). Some studies corrected for changes in the above BP (n = 22, 29%), HR (n = 21, 28%), or EtCO_2_ (n = 10, 13%) data in their analyses.

TCD protocols for continuous CBv measurements also differed significantly between studies, specifically with reference to unilateral versus bilateral measurements. Most studies performed bilateral insonation (n = 59, 78%), while a minority employed unilateral insonation (n = 11, 14%). Six studies (8%) did not report the insonation method. Overall, there was discordance among TCD insonation methods and concurrent measurements of BP, EtCO_2_ and HR.

For NVC protocols, selection of insonation vessel is an important consideration dependent on the brain area stimulated, and vessel in which a corresponding CBv increase should occur.^20^ Vessel monitored altered between MCA (n = 46, 61%), ACA (n = 2, 3%) or PCA (n = 5, 7%), individually or a combination of multiple vessels (n = 21, 28%) (Table 1). One study (1%) did not report the vessel insonated. The most commonly insonated vessel was the MCA, with 67 studies (88%) monitoring it individually or in tandem with other vessels. Few studies reported or controlled for the vessel segment measured (n = 19, 25%). However, those studies that reported segments monitored: M1 (n = 11, 58%), P2 (n = 9, 47%), P1 (n = 6, 32%), M2 (n = 1, 5%) and A1 (n = 1, 5%) segments. Some studies monitored multiple vessels. A summary of the task and the corresponding arteries insonated is included below.

### Protocol characteristics

The number of stimulation cycles ranged from one singular event to 20 repeats, which was pre-specified in studies using neuropsychological test batteries (e.g., N-back task). Studies differed between single (n = 9, 12%) or block (n = 67, 88%) event design, with the limited number of single event protocols making comparisons challenging. The largest NVC responses were produced by studies employing block design formats.

Stimulation type also varied. Most studies included a form of visual stimulation (n = 62, 82%) (not always exclusively), while a minority used only auditory stimulation (n = 10, 13%). Just under half of studies (n = 34, 45%) used standard neuropsychological tests, including the Stroop colour word (n = 8, 11%) or N-back tasks (n = 7, 9%). All studies required open eyes during stimulation, but a minority (n = 19, 25%) required closed eyes during rest/recovery. All studies which required closed eyes at any point used visual stimulation. Studies employing visual stimulation produced the largest CBv increases with augmented %CBv increasing by up to ∼30% from baseline. Studies employing cognitive stimulation produced CBv increases by ∼25%. Studies employing auditory stimulation induced CBv changes of ∼15%. Multiple forms of stimulation within the same study were common, making ranking stimuli challenging.

### Measurement characteristics

More than half of included studies (n = 40, 52%) used TCD devices from DWL (Singen, Germany). The second most utilised TCD devices (n = 13, 17%) were Nicolet-Pioneer or Viasys Healthcare Inc. (Conshohocken, PA., USA), followed by (n = 9, 12%) Spencer Technologies (Redmond, WA, USA), and (n = 5, 6%) Multigon (Elmsford, NY, USA).

The variety of different primary sources cited within protocols indicates discordance among studies. Several notable references and/or guidelines were used to describe technical procedures of TCD examination. TCD was invented in 1982 by Aaslid et al.,^21^ and this group is referenced most frequently (n = 9, 12%) in the 1980- and 1990s when TCD became popular. After the spread of TCD technology the second most cited references are Ringelstein et al., (n = 5, 6%)^22^ and Willie et al., (n = 5, 6%).^23^ In many papers a reference is not cited (n = 12, 16%).

CBv baseline durations varied between 250 ms (incorporated into neuropsychological test protocol) and >five minutes, or were not reported (n = 12, 16%). Some common baseline durations prior to stimulus were one-five minutes (n = 18, 23%), 31–60 seconds (n = 15, 19%), five-30 seconds (n = 13, 17%), >five minutes (n = 10, 13) and <five seconds (n = 9, 12%). For visual tasks, six studies (8%) used a duration of >60 s, and four (5%) were <five seconds prior to stimulus. For cognitive tasks, many studies used a duration >31 seconds (n = 26, 34%), and nine (12%) studies ≤30 s. For all other tasks (motor, acoustic, multiple domain tasks, arithmetic, orthostatic etc.), baseline durations were spread over the above time intervals. One third of studies (n = 25, 33%) used different stimulation periods within the same protocol, often simultaneously varying stimulus type. A distinct time point measuring the outcome variable was predefined in 34 studies (44%). Typically, studies which employed baselines >60 s produced the most significant NVC responses. Stimuli duration ranged 125 milliseconds (short visual stimulation) to 195 seconds. Rest periods ranged from five seconds to four minutes, with a minority of studies (n = 6, 8%) employing varying rest durations, dependent on the type of stimulation used. Two studies (3%) did not report stimulation duration. The most significant NVC responses were generated by studies which employed stimulation durations >ten seconds, which allowed sufficient time for CBv increases, and rest durations ≥30 seconds which allowed sufficient time for baseline haemodynamic return.

#### PCA assessed alongside visual stimulation

Sixteen studies examined PCA responses to visual stimuli. Measurements were usually performed in the left PCA and (as a measure of suggested unspecified brain activation) in the right MCA. The depth of recording was given in minimal (n = 4, 25%) studies with a range 58–70 mm. Depth was not reported in most studies (n = 12, 75%). The majority (n = 13) insonated the P2 segment, with fewer insonating the P1 segment (n = 3). In the accompanying sixteen MCA recordings, depth was rarely mentioned (n = 1, 6%), or not reported (n = 15, 94%). Four (25%) studies specified the M1-segment and, in one (6%) study, the M2-segment.

#### MCA assessed alongside cognitive stimulation

In 46 studies the MCA response was evaluated using cognitive (n = 40, 78%) tasks. The M1 segment was the recording site in most investigations (the M2 segment once). Mean reported recording depths were 47–59 mm (range 41–65 mm). In a minority of studies, the depth was not reported. In a minority of studies, cognitive tasks were evaluated by ACA insonation (depth 55–70 mm) alone or additionally to the MCA.

#### Other forms of stimulation

In ten studies, distinct tasks were examined. Amongst them, motor tasks were most frequent (n = 5, 50%). Recording depth was not reported in any study. One study (10%) examined multiple domains without reporting recording depth. In four other studies (40%) (one acoustic task, one arithmetic task, one orthostatic task and one wording task) recording depth ranged 46–60 mm.

### Analysis characteristics

**Figure 3.**
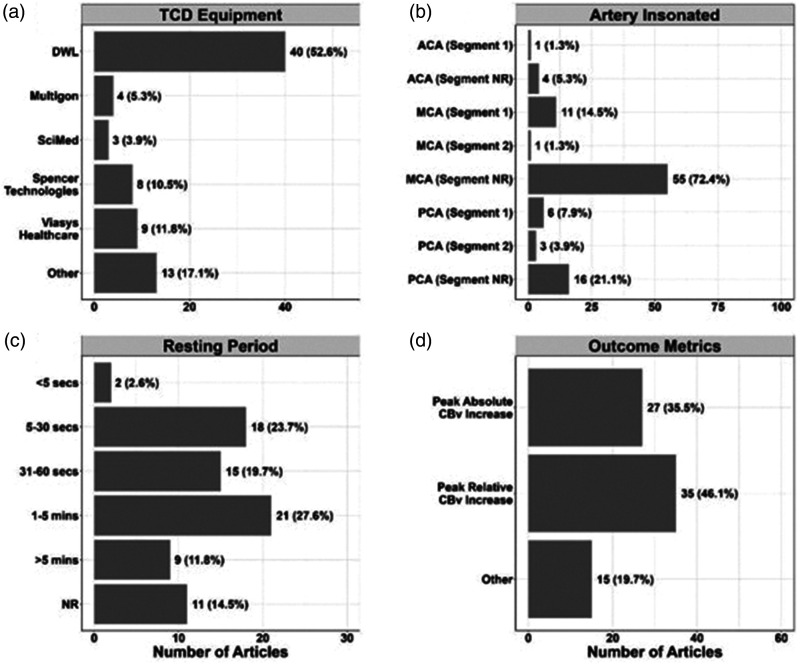
Summary of key study characteristics. a = TCD equipment used. b = Artery insonated. c = Resting period duration. d = Outcome metrics observed.

Common outcome measures were peak percentage change in CBv (n = 35, 46%) and absolute CBv change from baseline (n = 27, 36%) (Figure 3). Additional measures included the absolute CBv peak value or change in mean/systolic or diastolic CBv from baseline (n = 18, 24%), change in median CBv (n = 3, 4%), area under CBv curve (AUC) (n = 9, 12%), and time-to-peak CBv (n = 4, 5%). Additional outcome measures such as CBv peak value (or peak percentage change from given baseline), AUC and time-to-peak CBv provided complementary information of NVC responses but were infrequently reported together. Less commonly reported outcomes included: task-to-baseline CBv ratio (n = 1, 1%), CBv pulsatility index (n = 7, 9%), change in CBF (n = 4, 5%) (obtained from CBv accounting for vessel diameter), resistance area product (VRAP) (n = 4, 5%) and cerebrovascular resistance changes (CVR) (n = 10, 13%) (both aim to account for the effects of BP changes on CBv). Critical closing pressure (CrCP) (n = 3, 4%) and CBv spectral power (n = 2, 3%) were also reported. Several studies used lateralization-based indices (n = 11, 14%) and lateralization index variability (n = 1, 1%). In addition to CVR or VRAP to account for effects of BP on CBv, some studies investigated interplay of NVC mechanisms to pressure autoregulation (n = 12, 16%), baroreflex sensitivity (n = 1, 1%), and cerebrovascular reactivity assessed by CBv responses to CO_2_ changes (10, 13%). However, there was largescale inconsistency among reporting, hindering cross-study statistical analyses/comparisons and reproducibility assessments and adjustment may be necessary.

Several studies examined effects of different physiological conditions, alongside physiological manipulations on NVC. The most frequently examined conditions were aging (n = 11, 14%), and sex (n = 6, 8%). Other factors included cognition. Physiological manipulations included: hypercapnia and hypoxia (n = 3, 4%), hyper/hypoventilation (n = 1, 1%), high altitude (n = 2, 3%), orthostatic stress (n = 3, 4%), exercise (n = 3, 4%), sleep deprivation (n = 1, 1%), resveratrol (a phytoestrogen) hormonal therapy (n = 1, 1%), nitrate supplementation (n = 1, 1%), and cyclooxygenase inhibition (n = 1, 1%). Typically, aging and sex induced contradictory results upon NVC with studies showing increased, decreased, and neutral effects of both. Hypoxia was concluded to have no significant effect on NVC in included studies, however hypercapnia induced a reduction in peak CBv and NVC. High altitude and nitrate supplementation both had no significant effect on NVC. However, sleep deprivation reduced NVC, and cyclooxygenase inhibition reduced CBv, while hormonal therapy (resveratrol) induced larger NVC responses.

Further variation was found in reporting peak, mean systolic BP and end-diastolic BP. 24 studies (32%) reported peak systolic BP, while 32 (42%) reported mean systolic BP and 33 (43%) reported end-diastolic BP. All studies reported additional outcomes which were study-specific and were often related to other factors including: cognitive test scores, demographic data and fNIRS data.

Four studies (5%) considered CBv power spectrum. One study (1%) investigated relationships between memory performance and CBv power spectrum with and without additional disturbance induced by lower body negative pressure oscillations. One study (1%) examined differences in power distribution between males and females during cognitive tasks. Two studies (3%) used transfer function analysis (TFA) to determine frequency domain metrics of cerebral autoregulation (CA), i.e., the dynamic regulation between arterial BP and CBF. One study (1%) investigated relationships between vasomotor reactivity to CO_2_ and NVC, evaluated with time-domain and TFA metrics calculated at rest. One study (1%) evaluated TFA metrics during mental tasks.

Four studies (5%) reported implementing autoregressive–moving-average (ARMA) modelling. ARMA modelling was used to separate the effects of CA and CO_2_ reactivity from the main effects of cognitive-motor stimulation, to obtain spectral estimates, including partial pressure of carbon dioxide (PCO_2_) in bispectral analysis and in the pre-whitening of data. One study (1%) conducted logistic regression analysis to reveal potential predictors of task performance.

### Reproducibility measures

Twenty-seven studies (36%) undertook reproducibility assessments of their protocol, while 65 studies (86%) referenced previously validated protocols which were incorporated into their methods, citing a measure of reproducibility. Five studies (7%) reported intraclass correlation coefficients (ICC) while eight (11%) reported coefficient of variance (CoV), and 21 studies (28%) reported standard error of mean (SEM). ICC ranged from <0.4 to >0.9, CoV was low (2.54/4.9%) and SEM ranged from 2% to 209%.

Of the included studies, only four assessed either between-day or within-day reliability (completing repeated testing in the same individuals using the same paradigm, with various statistical approaches). These approaches included ICC (n = 4, 5%), CoV (n = 4, 5%), Bland-Altman (BA) plots (n = 1, 1%), SEM (n = 1, 1%), and/or typical error of measurement (n = 1, 1%). Cognitive tasks were used in two of these investigations (modified incongruent Stroop task and Addenbrooke’s Cognitive Examination III (ACE-III)^24^) while a visually evoked response was used in another (“Where’s Waldo?” task). The MCA was insonated in all four studies, while the common carotid artery (CCA) and PCA were additionally insonated within the modified Stroop task and visual task, respectively. The four studies which assessed reliability produced mixed results. Some results indicated that CBv responses to the ACE-III were variable, but that AUC may be more dependable than peak CBv for measures of NVC. Another study reported that NVC responses demonstrated excellent within-day reliability in ≥5 trials. A further study reported most NVC metrics displayed excellent reliability (>0.90), except time‐to‐peak PCAv, which resulted in reduced within‐day and between‐day reliability (<0.50). A final study only performed a between-day analysis in a small subset of the cohort, limiting conclusions that could be drawn.

## Discussion

### Summary of results

Included studies encompassed a variety of characteristics (Table 1). Multiple postural positions were incorporated including seated, supine, and walking. Most studies insonated the MCA. Most studies included simultaneous assessments of BP, HR and EtCO_2_. Many studies employed varying stimulation durations and types. Similar trends were seen with rest periods. Baseline durations were most common between the one-five-minute window. Reproducibility assessments were seen infrequently, but most studies incorporated previously validated protocols into their methods. There was an even spread of baseline periods, while minimal ARMA, TFA and logistic regression analyses were reported. Overall, characteristics associated with larger or more dependable NVC measurements included: seated posture, visual stimuli, stimuli durations >ten seconds and rest durations ≥30 seconds.

### Context and recommendations

#### Preparation

A sizeable proportion of studies employed a seated position for participants compared with other postures, which was associated with larger, more significant NVC responses. This finding mirrors that of previously published work. McDonnell et al.,^25^ showed that vasomotor reactivity assessed by TCD was significantly increased when assessed in healthy participants in a seated position compared to participants in other conditions such as supine. However, some protocols may use a specific postural condition due to protocol limitations or to answer specific research questions, so this finding may not be relevant to all studies/protocols.

Many studies in this review used visual stimuli (e.g., light flashes, colour changes or checkerboard) to induce NVC responses.^17,26^ Study environment light intensity control may be beneficial in visual stimulation protocols, as the small cohort of included studies which did control light intensity produced larger NVC responses. This may strengthen NVC responses by limiting light exposure prior to stimulation.

The small proportion of studies which reported time of day may be linked to the quality of study reporting. A recent study by Burma et al., (2021) specifically assessed how time of day affects NVC and found no temporal variation in NVC metrics throughout the day.^27^ However, this may be dependent on the stimuli being used (‘*Where’s Waldo?’*) and other stimuli may provide contradictory results. This requires further investigation in future studies using different NVC stimuli.

Few studies controlled for food consumption prior to NVC assessments. This may vary by the size of the meal, and length of time prior to the assessment, but the literature suggests a fasted state has minimal impact on CBF.^28^ In addition, the most significant NVC responses were recorded in both studies that did and did not control for food consumption beforehand.

*Recommendations:*

1. *The seated position is recommended to optimise NVC assessments, unless the study protocol is investigating different postural conditions, or the protocol precludes a seated assessment.*
2. *Controlling for environmental light intensity or using an eyes closed/open protocol is recommended for visual stimulation studies.*
3. *Current evidence suggests time of day does not affect NVC responses, but further work is needed to clarify this across more stimulation types and cohorts.*
4. *Fasted states do not affect NVC responses in this review and may not be necessary to control for NVC studies.*
5. *A uniform reporting policy would better represent the effects of different stimuli in different study environments.*

#### Assessment characteristics

Most included studies insonated the MCA. This approach may not be ideal as varying the artery insonation assesses alternative vessels supplying different cortical regions. For example, NVC elicited through visual stimulation activates the visual cortex via the PCA^29^ which supplies the occipital lobe,^30^ while the MCA is associated with cognitive processing and supplies ∼80% of anterior and lateral aspects of the brain.^31^ However, visual stimuli can also elicit small MCA CBv changes.^32^ Therefore, combined approaches using multiple vessels (notably MCA and PCA) and stimulation types will provide more informative NVC assessments.

Despite minimal implementation, concomitant high resolution, beat-to-beat/breath-by-breath peripheral measurements (BP, HR and EtCO_2_) allow for control/correction of NVC results in analysis, as all three parameters can confound NVC measurements. For example, Bathala et al., showed HR has potential to influence various flow parameters (including CBv), which could impact NVC respones.^33^ Manno and colleagues showed induced hypertension and BP alteration did affect CBv and CA.^34^ Webb et al., also showed EtCO_2_ had potential to impact CBv responses in stroke populations but required concurrent beat-to-beat BP measurements.^35^ However, in studies included in this review, CBv responses did not differ between those that did and did not correct for these variables. Concomitant measurement of these parameters is standard in CA research and recommended in the CARNet White Paper.^36^

*Recommendations:*

1. *Multi-vessel and multi-task approaches are recommended to optimise assessments and provide a more comprehensive summary of NVC response in different brain regions. Specifically, complementary task, and vessel assessments are recommended, such as PCA insonation for visual stimulation.*
2. *Concomitant high resolution, beat-to-beat/breath-by-breath BP, HR and EtCO_2_ measurements are recommended where possible to facilitate correction for confounding factors in NVC analysis.*

#### Protocol characteristics

The stimulation durations varied according to the type of stimulation used. For example, some studies used tasks with standardised durations, such as the N-back task, which uses multiple averaged four-second stimulation periods as a validated measure of working memory.^37^ The N-back protocol was used by several studies in this review.^38,39^ However, the stimulation period may also vary within protocols because specific test batteries have pre-determined durations, which may be suitable for particular cohorts.^40^ For example, the ACE-III was used in one study^13^ which has both varying stimulation types and durations as part of a standard neuropsychological test for cognitive impairment. However, stimuli <ten seconds had insufficient time to generate significant haemodynamic responses. Rest period durations also varied and were consistent with literature suggesting extended rest periods allow haemodynamic signals to return to baseline, notably in durations ≥30 seconds.^41^ However, the N-back task also has pre-determined rest period durations, limiting flexibility to adjust this. Typically, baseline durations prior to the stimulation protocol were between one and five minutes. However, extended baseline durations typically enable more accurate averaged baseline haemodynamic data collection, meaning the higher reaches of this period should be targeted.^42^

Few studies evaluated reproducibility of NVC assessments. However, novel methods have use as they can be custom-built for specific research questions, but these require concomitant reproducibility and reliability testing. A minority of studies completed within- and between-day analyses. While numerous techniques are used to determine NVC responses, little is known regarding the specific reliability of these techniques using TCD. Comparatively, meta-analyses of 90 studies have been completed on BOLD-fMRI literature during task engagement.^43^ Ultimately, using a technique with an unknown between- and/or within-day reliability poses the risk of mitigating the internal validity of a repeated-measures study design. This is because it would remain unclear whether temporal changes are due to a clinical condition, natural variability, or experimental intervention. However, it is important to consider there are larger relative increases for PCAv for visual task^26,44,45^ as compared to MCAv for cognitive stimulation^24,46^ tasks, which may underlie the higher reliability seen for visual stimulation in the PCA.

*Recommendations:*

1. *Stimulation periods of a minimum duration of >ten seconds are recommended to allow sufficient CBv increase in response to stimulation alongside rest period durations* ≥*30 seconds to allow baseline haemodynamic return.*
2. *Baseline periods >one minute in duration are recommended to ensure accurate NVC assessments are recorded.*
3. *More work is recommended investigating reproducibility and reliability of different stimulation types and protocols. This includes investigation of between- and/or within-day reliability assessments.*

#### Measurement characteristics

Recommendations for a single TCD device would be inappropriate but some characteristics shared by commonly used devices should be accounted for. Comparison between non-duplex and duplex devices indicates arteries are blindly identified in non-duplex methods, highlighting possible needs for transition to widespread duplex TCD usage,^47^ although current availability remains limited. Duplex TCD (for example, specifically, Transcranial colour-coded Sonography, which colour codes CBv allowing basal cerebral artery visualisation) increases accuracy and acceptability of TCD protocols^47^ by aiding vessel location. However, beat-by-beat Transcranial colour-coded Sonography CBv outputs are limited, providing poorer temporal resolution and significantly limiting application for NVC assessments, particularly in combination with other high-resolution outputs including beat-to-beat BP.

Several notable references and/or guidelines were used to describe technical procedures of performing TCD examinations. These standard approaches may only be suitable for specific research protocols and lack flexibility for novel approaches. Novel approaches may be required to adjust TCD-based NVC assessments for specific patient groups and clinical applications.^48^ For example, standard approaches may be intolerable to those with cognitive impairment and techniques may require adaptation to insonate different arteries (MCA, PCA, ACA) or through different windows (e.g., transorbital window, suboccipital window). Few studies reported segments or depths insonated, which would strengthen study reporting and consistency between future studies.

*Recommendations:*

1. *Studies should reference previously validated or published guidelines, or assessments of reproducibility where possible, accepting that novel approaches may be required to tailor to specific populations.*
2. *Use of duplex TCD with high, beat-by-beat temporal resolution, where available, is recommended to enhance accuracy in identifying vessels and segments for insonation.*
3. *Standardised reporting of vessel segments and depth insonation is recommended to improve consistency between studies.*

#### Analysis characteristics

##### Time-domain assessment of neurovascular coupling

A range of analysis characteristics were employed, but most studies assessed the peak or average percentage change in CBv from baseline. Reporting additional measures of CBv peak value, AUC, and time-to-peak CBv provided a more comprehensive summary of NVC in included studies. It is evident that many time-domain measures have been used, but it is also unclear which measure is most sensitive and robust in quantifying NVC changes in different populations or conditions, due to differing implementations and outcome metrics. One analysis performed a small comparison of linear and non-linear techniques and time-domain NVC assessments and addressed some limitations using fNIRS rather than TCD-based assessments.^49^ A similar approach may be beneficial for TCD studies.

#### Combined analysis approaches

Combined analysis approaches were scarce.^50^ One study employed a number of analyses using a large NVC dataset of more than 100 people, where most known time-domain metrics of NVC were determined for each individual.^29^ Computational approaches were used that included iterative dimensionality reduction to reduce redundancy in the metrics, and principal component analysis to evaluate associations between the substantial number of variables. Clustering was used to identify similar variables within and between individuals, and benchmark/validation testing by age, sex and between clinical conditions, although no specific metrics were recommended over others. Another study used principal component analysis to identify the major contributors to task-activated neurovascular responses,^51^ which identified twelve significant factors accounting for 78% of the variation. Peak percentage change in CBv and visuospatial stimulation accounted for a majority of the variance identified. One included study employed a similar approach to understand how eye movement patterns influence the NVC response, showing that task complexity, eye movement speed, and eye movement amplitude contributed to NVC response magnitude.^50^ However, another study assessed scalability of common paradigms and found no effect of task complexity or duration.^51^ However, due to few studies involving these approaches, minimal conclusions regarding optimal analysis approaches for TCD-based NVC assessments can be drawn.

##### Dynamic linear model analysis

Few studies (n = 2, 3%) examined NVC using dynamic linear modelling,^7,26^ each using different methods, concluding that myogenic regulation dominates autoregulatory responses and that ABP dominates V_RAP_ (myogenic pathways) in models that fit NVC responses well. Methods used included lumped parameter models of the PCA, second-order linear systems explaining peak percentage CBv change, ARMA modelling assessing distinct transient effects of ABP and EtCO_2_ on CBv during external neuronal stimulation, and second-order dynamic systems obtaining systolic and diastolic CBv changes. In addition, three other studies (4%) employed autoregressive dynamic linear methods solely for data preprocessing and visualisation purposes, concluding that oscillatory CBF induces NVC reductions in response to a memory task.^52^ However, implementation is regrettably restricted, meaning the various methods and outcomes require further investigation, despite promising results.

##### Frequency domain

Few studies also considered the power spectrum of CBv (n = 2, 3%),^52,53^ relationships between memory performance and CBv power spectrum (n = 1, 1%),^52^ and differences in power distribution between males and females during cognitive tasks (n = 1, 1%).^53^ These studies concluded that lower body negative pressure raised oscillatory power at oscillation frequencies not suppressed by a memory task and that NVC responses do differ by sex as female lateralisation shifted from right to left hemispheric dominance, respectively. There was also minimum exploration of TFA^36^ specifically to compute frequency domain metrics of CA and relationships between vasomotor reactivity and NVC at rest and during mental tasks (n = 1, 1%). Again, this limits conclusions that can be drawn, and further investigation is required for between-study analyses. However, a majority of the field implements time domain measures, which provide many opportunities to assess parameter effects on NVC in different populations and conditions and aids downstream analyses with stable signals.

*Recommendations:*

1. *The reporting of outcome measures such as CBv peak value (or peak percentage change from given baseline), AUC and time-to-peak CBv is recommended in addition to absolute and peak percentage change in CBv, to provide a comprehensive overview of NVC responses.*
2. *Further investigation (sensitivity analysis) into alternative approaches is recommended including which of absolute CBv changes and relative CBv changes is more robust in quantifying NVC changes in different populations or conditions. This should be addressed in a systematic manner by comparing outcomes obtained by different measures on the same dataset.*
3. *Further research using dimensionality reduction, principal component analysis and clustering are recommended to help identify the most useful metrics for TCD-based NVC assessments.*
4. *Further investigation of approaches such as dynamic linear modelling and ARMA analysis is recommended to understand their utility in TCD-based NVC assessments, provided data is of sufficient resolution for accurate analyses.*
5. *Further investigation into the utility of frequency domain analysis is recommended, provided relevant data is of sufficient resolution for accurate analyses.*

#### Clinical applications of NVC

NVC has been investigated using TCD in a number of clinical populations, some of which have been the subject of previous reviews.^54^ Despite the scope of this review assessing primarily healthy TCD-assessed NVC, we do acknowledge key diseases that have been investigated using TCD-measured NVC approaches.

##### NVC in cardiovascular disease

Cardiovascular disease can influence the health of vital organs and influence NVC.^55^ Visually evoked increases in PCA CBv are impaired in patients with systemic hypertension,^55^ coronary artery disease,^56^ coarctation of aorta,^57^ reduced ejection fraction heart failure^58^ and atrial fibrillation.^59^ Hypertension and comorbid diabetes result in further deterioration of the NVC.^55^ However, NVC differences may be confounded by differences in resting CBv between patients with hypertensions and healthy controls.^60^ This group recognises requirements for exploration of therapies addressing primary cardiovascular pathology. This exploration may include accompanying pathophysiological alterations like endothelial dysfunction, and/or treatment of associated comorbidities, effectively rescuing the NVC response.

##### Motor NVC in stroke

An active or a passive motor task is typically accompanied by a bilateral CBv increase in both MCAs.^61^ In healthy participants, responses are dependent on BP and EtCO_2_.^62^ Imagination of a motor task also leads to a bilateral CBv increase but to a lesser extent.^63^ NVC is shown to also be disturbed in a severity-dependent manner, and correlates to functional motor recovery three months post-stroke.^64^

##### NVC in dementia and cognitive disorders

NVC has been investigated to a greater extent in mild cognitive impairment (MCI) and Alzheimer’s disease (AD), using a range of stimuli. The most common approaches have used visual stimuli such as checkerboards/light^65^ and cognitive tasks.^13^ In comparisons between AD and vascular dementia sub-types, NVC is affected to a greater degree in vascular sub-types.^65^ In most studies, NVC is depressed in dementia^65^ and, to a lesser degree, in MCI.^66^ However, baseline CBv may be also reduced in dementia patients, potentially confounding reduced CBv responses to cognitive stimuli.^67^ However, MCI can show variable patterns of activation, with both increases and decreases in CBv in response to activation.^54^ This may be due to the stage of MCI, with those at earlier stages demonstrating increased CBv responses to compensate for declining neuronal and cognitive function, and those at latter stages demonstrating a failure of these compensatory mechanisms.^54^ Also, depressed NVC has been shown to be associated with slower walking and diminished locomotor control in elderly people.^68^

##### Traumatic Central nervous system injury and NVC

NVC changes following traumatic brain injury (TBI) have been limited to patients with mild or moderate TBI. Inability to follow commands such as reading or visual scanning limits the ability to assess NVC in patients with severe TBI. However, a growing body of data is elucidating changes in CA following TBI, especially in patients with severe TBI.^69^ Measuring CBv in the PCA with TCD before and following a visual stimulus has been used in mild and moderate TBI patients.^45^ In a single-centre study of contact sport athletes, NVC was assessed pre-season and following concussion at three days, two weeks, and one month. NVC was measured by PCA CBv changes in response to a visual stimulus. The response magnitude of PCA velocity increased 50% at three days following concussion, which persisted at the two-week post-concussion evaluation. These changes in NVC at two weeks persisted despite return of cognitive performance to pre-injury baseline and resolution of self-reported symptoms.^70^ In people with spinal cord injury and the associated hypotension, CBv responses are reduced to cognitive tasks compared to healthy controls and are associated with cognitive impairments.

##### Subarachnoid haemorrhage and NVC

There is limited clinical data of NVC changes following subarachnoid haemorrhage (SAH) due to impaired ability of patients to fully participate in assessments. Furthermore, care standard in treating SAH is administering nimodipine (dihydropyridine calcium channel antagonist),^71^ affecting contraction of vascular smooth muscle and confounding cerebral vascular responses. Animal models without confounding factors may provide insights into NVC changes following SAH. Two-photon excitation microscopy provides real-time *in-vivo* measurements of NVC by measuring cerebral vasculature diameter and CBF following tactile stimulation.^72^ Mice subjected to Circle of Willis filament perforation inducing SAH were compared to shams exposed to filament insertion into the common carotid artery with no arterial rupture. Pial and parenchymal arteriole diameter and CBF over the contralateral somatosensory cortex were measured following forepaw stimulation. Arterioles in mice following SAH did not dilate in response to forepaw stimulation compared to sham mice. CBF also did not change following forepaw stimulation compared to sham mice.^42^ These data demonstrate impaired NVC 30 days after SAH in a mouse model.

*Recommendations:*

1. *The exploration of NVC in cardiovascular pathology and novel therapies is recommended, including any associated comorbidities to effectively rescue the NVC response.*
2. *Adjustment of baseline CBv in analyses to eliminate confounding in observed NVC differences between healthy and disease states is needed.*
3. *Further investigation into anterior and posterior circulation infarcts, using cognitive stimuli is recommended.*
4. *Further investigation into NVC as a potential biomarker and therapeutic target for neurological and cognitive disorders is recommended.*
5. *Further investigation is recommended into the utility of NVC in SAH and TBI using non-demanding motor or sensory stimuli, building on the work of mouse models.*

#### Multimodal neuroimaging

TCD-based measures of NVC are not without limitation, and, despite being outside the scope of this review, multimodal approaches can be advantageous to remedy TCD shortcomings. It is important to consider the nature of TCD-based global flow measures and the magnitude of stimuli required to induce measurable global blood flow changes. For instance, studies combining imaging approaches with TCD (e.g., fNIRS) illustrate that regional CBF changes within the brain are not necessarily reflected in the conduit vessel which supplies that region.^73^

In this context, alternative non-invasive techniques have also been used for obtaining proxy *in-vivo* measurements for regional CBF, including fMRI and fNIRS. These imaging techniques provide CBF measurements at higher (∼2–3 mm) spatial resolutions than TCD, albeit with certain caveats. Among various fMRI techniques used for measuring indices of CBF changes, including perfusion^74^ and phase-contrast MRI,^75^ BOLD-fMRI is the most widely used non-invasive technique for measuring changes in brain activity during task performance or the resting-state.^76^ The BOLD-fMRI signal is sensitive to dynamic changes in the oxygenated:deoxygenated haemoglobin ratio,^76^ which is coupled to the underlying neuronal activity and subsequent spatial and temporal changes in CBF via NVC mechanisms.^77^ Similar principles underlie fNIRS where near infrared light penetrates the brain via optodes placed onto the skull, quantifying the BOLD-based haemoglobin oxygenation state within the cortical cerebrovascular network of interest.^78^ However, it should be noted that BOLD-fMRI and fNIRS do not provide a direct quantitative measurement of CBF; this can be achieved by using arterial spin labelling (ASL) fMRI.

fNIRS, specifically may be used in a complementary manner to TCD, with TCD quantifying CBv within the Circle of Willis, whereas fNIRS provides haemoglobin oxygenation states at a more superficial level.^78^ This would provide a more comprehensive overview of cerebral haemodynamics in more brain areas than these techniques could reach individually, as was employed by Lefferts et al.,.^79^ BOLD-fMRI may also be suitable for integration with a TCD approach. As stated, TCD is subject to poor spatial resolution,^7^ which may be mediated by the increased spatial resolution of BOLD-fMRI. However, due to practical difficulties, these techniques may need to be performed at contrasting times, limiting the data that can be drawn as data cannot be compared.^77^ These practical challenges explain minimal implementation of this integrated approach.

ASL and BOLD-fMRI can be acquired concurrently, which may provide unique information for the assessment of NVC via estimates of parameters of cerebral physiology, such as the rate of oxygen metabolism.^80^ PET has also been used for assessing CBF and NVC.^81^ Still, due to the requirement for radiotracers, the inflated cost associated with radiotracer production, and the low temporal resolution of the technique, PET imaging has only been used on a limited basis compared to fMRI and fNIRS to date.

While fMRI, ASL, and fNIRS provide valuable insights into global and regional CBF changes, assessing NVC using these imaging techniques typically requires external cognitive/sensory stimulation and precise knowledge of the timing of each stimulus.^82^ However, combining these imaging techniques with electrophysiological measurement allows for NVC investigation, even without external stimulation.^83^ The most common non-invasive method for measuring human *in vivo* neuronal activity is electroencephalography (EEG). EEG uses electrodes attached to the scalp to detect subtle electrical potential fluctuations emerging from post-synaptic potentials changes along neocortical pyramidal cell apical dendrites.^84^ Simultaneous EEG-fNIRS integration may be coupled with TCD, provided practical difficulties can be overcome. Moritz et al.,^85^ trialled integration of these approaches and determined that all three efficiently assessed cerebral haemodynamics in small patient groups undergoing carotid surgery. EEG complimented the previously described combination of superficial and deep brain TCD-fNIRS by providing detailed overviews of corresponding electrical potentials to CBF variations within NVC.^85^

Interestingly, combined imaging techniques have uncovered discrepancies between local CBF measurements derived from BOLD-fMRI and increases in glucose metabolism observed in functional PET imaging.^86^ This suggests CBF changes may not fully correspond to synaptic activity, highlighting the need for further investigation and understanding of underlying NVC mechanisms. The wide range of multimodal imaging and analysis protocols renders comparing results from different studies challenging. It remains unclear whether observed differences in study findings are due to spontaneous physiological variation (BP, O_2_ & CO_2_ fluctuations), differing populations studied, or methodological differences, limiting translational application.

*Recommendations:*

1. *Further investigation into more informed assessments of NVC using multimodal approaches are recommended. These measurements should be guided by data from other imaging modalities, remedying TCD shortcomings, including poor spatial resolution. Investigations into inconsistencies such as those between BOLD-fMRI and PET imaging glucose metabolism are also recommended integrating multiple imaging modalities to better understand NVC mechanism physiological variance.*

### Strengths, limitations, and future work

This review used a comprehensive search strategy to synthesise literature on TCD-based NVC methods in healthy populations and used an international consensus group to draw recommendations for future NVC studies using TCD and identified gaps for future research. This will facilitate alignment of future NVC protocols and provide areas for currently unanswered research questions to stimulate future NVC research with TCD.^87^ This review is limited to TCD-based assessments of NVC in healthy populations. It was beyond the scope of this review to examine all clinical populations and other methods to assess NVC, but these are summarised in the discussion. There was significant heterogeneity between included studies, specifically: protocol design, stimulation type, baseline and stimulation durations, and vessel studied, thereby preventing meta-analyses. Future work should extend this review to clinical populations and complementary imaging modalities (e.g., fNIRS).

### Conclusions

This review provides a set of recommendations to standardise TCD-based NVC assessments in healthy populations, and highlights gaps for future research to optimise NVC methods using TCD. Despite limitations surrounding included studies, we have determined key protocol and measurement characteristics that can be optimised for future NVC research. Further investigation is required into different cohorts, complementary techniques, and analyses for further optimisation of TCD-based NVC assessments.

## Supplemental Material

sj-pdf-1-jcb-10.1177_0271678X241270452 - Supplemental material for Neurovascular coupling methods in healthy individuals using transcranial doppler ultrasonography: A systematic review and consensus agreement Supplemental material, sj-pdf-1-jcb-10.1177_0271678X241270452 for Neurovascular coupling methods in healthy individuals using transcranial doppler ultrasonography: A systematic review and consensus agreement by James D Ball, Eleanor Hills, Afzaa Altaf, Pranav Ramesh, Matthew Green, Farhaana BS Surti, Jatinder S Minhas, Thompson G Robinson, Bert Bond, Alice Lester, Ryan Hoiland, Timo Klein, Jia Liu, Nathalie Nasr, Rehan T Junejo, Martin Müller, Andrea Lecchini-Visintini, Georgios Mitsis, Joel S Burma, Jonathan D Smirl, Michael A Pizzi, Elsa Manquat, Samuel JE Lucas, Karen J Mullinger, Steve Mayhew, Damian M Bailey, Gabriel Rodrigues, Pedro Paulo Soares, Aaron A Phillips, Prokopis C Prokopiou and Lucy C Beishon in Journal of Cerebral Blood Flow & Metabolism

sj-pdf-2-jcb-10.1177_0271678X241270452 - Supplemental material for Neurovascular coupling methods in healthy individuals using transcranial doppler ultrasonography: A systematic review and consensus agreement Supplemental material, sj-pdf-2-jcb-10.1177_0271678X241270452 for Neurovascular coupling methods in healthy individuals using transcranial doppler ultrasonography: A systematic review and consensus agreement by James D Ball, Eleanor Hills, Afzaa Altaf, Pranav Ramesh, Matthew Green, Farhaana BS Surti, Jatinder S Minhas, Thompson G Robinson, Bert Bond, Alice Lester, Ryan Hoiland, Timo Klein, Jia Liu, Nathalie Nasr, Rehan T Junejo, Martin Müller, Andrea Lecchini-Visintini, Georgios Mitsis, Joel S Burma, Jonathan D Smirl, Michael A Pizzi, Elsa Manquat, Samuel JE Lucas, Karen J Mullinger, Steve Mayhew, Damian M Bailey, Gabriel Rodrigues, Pedro Paulo Soares, Aaron A Phillips, Prokopis C Prokopiou and Lucy C Beishon in Journal of Cerebral Blood Flow & Metabolism

sj-pdf-3-jcb-10.1177_0271678X241270452 - Supplemental material for Neurovascular coupling methods in healthy individuals using transcranial doppler ultrasonography: A systematic review and consensus agreement Supplemental material, sj-pdf-3-jcb-10.1177_0271678X241270452 for Neurovascular coupling methods in healthy individuals using transcranial doppler ultrasonography: A systematic review and consensus agreement by James D Ball, Eleanor Hills, Afzaa Altaf, Pranav Ramesh, Matthew Green, Farhaana BS Surti, Jatinder S Minhas, Thompson G Robinson, Bert Bond, Alice Lester, Ryan Hoiland, Timo Klein, Jia Liu, Nathalie Nasr, Rehan T Junejo, Martin Müller, Andrea Lecchini-Visintini, Georgios Mitsis, Joel S Burma, Jonathan D Smirl, Michael A Pizzi, Elsa Manquat, Samuel JE Lucas, Karen J Mullinger, Steve Mayhew, Damian M Bailey, Gabriel Rodrigues, Pedro Paulo Soares, Aaron A Phillips, Prokopis C Prokopiou and Lucy C Beishon in Journal of Cerebral Blood Flow & Metabolism
