# Supplementary material for: Neurovascular coupling methods in healthy individuals using transcranial doppler ultrasonography: A systematic review and consensus agreement
Source: J Cereb Blood Flow Metab. 2024 Aug 7;44(12):1409–29. doi: 10.1177/0271678X241270452 (PMC11572172; doi:10.1177/0271678X241270452)
Supplement: sj-pdf-1-jcb-10.1177_0271678X241270452 - Supplemental material for Neurovascular coupling methods in healthy individuals using transcranial doppler ultrasonography: A systematic review and consensus agreement [file sj-pdf-1-jcb-10.1177_0271678X241270452.pdf]

1. "Cognitive stimul*".mp.
2. "Cognitive activation".mp.
3. "Neurovascular coupling".mp.
4. NVC.mp.
5. "Cerebral stimulation".mp.
6. "cerebral activation".mp.
7. "motor stimul*".mp.
8. "verbal stimul*".mp.
9. "auditory stimul*".mp.
10. sound*.mp.
11. "task activation".mp.
12. "tactile stimul*".mp.
13. "acoustic stimul*".mp.
14. noise.mp.
15. "Healthy TCD".mp.
16. "Healthy Transcranial doppler".mp.
17. "transcranial doppler".mp.
18. "trans cranial doppler".mp.
19. "transcranial ultrasound".mp.
20. "trans cranial ultrasound".mp.
21. "transcranial ultrasonography".mp.
22. "trans cranial ultrasonography".mp.

23. "transcranial sonography".mp.

24. "trans cranial sonography".mp.

25. TCD.mp.

26. "Functional neuroimaging".mp.

27. "functional neuro imaging".mp.

28. "functional brain imaging".mp.

29. 1 or 2 or 3 or 4 or 5 or 6 or 7 or 8 or 9 or 12 or 13 or 14

30. 15 or 16 or 17 or 18 or 19 or 20 or 21 or 22 or 23 or 24 or 25 or 26 or 27 or 28

31. 29 and 30

32. limit 31 to humans
