# Supplementary material for: Neurovascular coupling methods in healthy individuals using transcranial doppler ultrasonography: A systematic review and consensus agreement
Source: J Cereb Blood Flow Metab. 2024 Aug 7;44(12):1409–29. doi: 10.1177/0271678X241270452 (PMC11572172; doi:10.1177/0271678X241270452)
Supplement: sj-pdf-2-jcb-10.1177_0271678X241270452 - Supplemental material for Neurovascular coupling methods in healthy individuals using transcranial doppler ultrasonography: A systematic review and consensus agreement [file sj-pdf-2-jcb-10.1177_0271678X241270452.pdf]

Study published in peer-reviewed journal

Aims/hypothesis of research is described in the introduction/methods

The population of the study is described (age, sex, co-morbidities)

Study has ethical approval AND informed consent taken from study participants

Sample size calculated before start of experiment

Inclusion and exclusion criteria are clearly described

Study protocol clearly described

Statistical methods presented and appropriate

CBF measurements clearly presented and consistent

CBF(v) calculation is present

Graph/table summarising results

Study tested reliability and validity of used measurements or referred to other studies which established reliability and validity

Strength of evidence for each main outcome was discussed

Limitations of the study are presented and discussed

Suggestions made for future research

Authors' conflicts of interest are declared
