# Supplementary material for: Neurovascular coupling methods in healthy individuals using transcranial doppler ultrasonography: A systematic review and consensus agreement
Source: J Cereb Blood Flow Metab. 2024 Aug 7;44(12):1409–29. doi: 10.1177/0271678X241270452 (PMC11572172; doi:10.1177/0271678X241270452)
Supplement: sj-pdf-3-jcb-10.1177_0271678X241270452 - Supplemental material for Neurovascular coupling methods in healthy individuals using transcranial doppler ultrasonography: A systematic review and consensus agreement [file sj-pdf-3-jcb-10.1177_0271678X241270452.pdf]

Author

Year

Setting

No. of participants

Age

Sex

Comorbidities

Inclusion criteria

Exclusion criteria

Position

Time of day controlled

Quiet environment

Abstained from exercise

Abstained from alcohol

Abstained from large or heavy meals

BP recorded

$EtCO_2$ recorded

HR recorded

Unilateral or bilateral TCD

Stimulation period

Recovery period

No. of cycles

Type of stimulation

Previously validated

Reproducibility assessed

Description of paradigm

Description of control/rest phase

Duration of paradigm/stimulation

Eyes open/closed

Single event or block design

Vessel monitored

Segment monitored

Depth recording

Guidelines used/referenced for insonation

TCD device

Baseline duration

Time point for peak change

Main outcome

Peak systolic BP

Mean systolic BP

End diastolic BP

Additional outcomes e.g. AUC, time to peak, %change

Main findings, quantify level of % change

ARMA

TFA

Logistic regression

Intraclass Correlation Coefficient (ICC)

Coefficient of Variance (CoV)

Standard Error of the Mean (SEM)

Notes
